# An Unusual Superficial Neck Abscess Confined to the Skin and Subcutaneous Tissue: A Case Report

**DOI:** 10.7759/cureus.117994

**Published:** 2026-10-15

**Authors:** Arın Celayir

**Affiliations:** 1 Department of Orthopaedics and Traumatology, Çukurca State Hospital, Hakkari, TUR; 2 Department of Orthopaedics and Traumatology, Cerrahpaşa Faculty of Medicine, Istanbul University-Cerrahpaşa, Istanbul, TUR

**Keywords:** drainage and debridement, neck abscess, s. aureus, staphylococcus aureus, superficial neck infection, surgical drainage

## Abstract

A cervical abscess, or an abscess in the neck region, is a localized pus buildup resulting from an infection. It usually develops when bacteria enter the tissues through a skin break, throat infection, or dental problem. Common symptoms include pain, swelling, redness, and warmth in the affected area, and it may be accompanied by fever and difficulty swallowing or breathing if the abscess is large or compresses nearby structures. Treatment typically involves antibiotics to combat the infection and surgical drainage to remove the pus, alleviating pressure and preventing further complications. In this report, we have described the case of a patient who presented with swelling and discharge on the skin of the nape, was diagnosed radiologically with an abscess, and subsequently underwent surgical treatment.

## Introduction

A skin and soft-tissue abscess is a localized collection of purulent material within the dermis or subcutaneous tissue. Pain, swelling, erythema, warmth, fluctuance, and spontaneous drainage are common findings, although systemic manifestations may be absent. *Staphylococcus aureus*, including methicillin-resistant strains in some settings, is a leading cause of purulent skin and soft-tissue infection [1-3].

For a clinically significant abscess, incision and drainage constitute the principal source-control procedure. Adjunctive antimicrobial therapy is individualized according to systemic findings, host factors, anatomic location, local resistance patterns, and microbiological results [1-4]. In the neck, establishing whether infection is limited to superficial tissues or extends into the deep cervical spaces is particularly important because deep extension may threaten the airway, spread along fascial planes, or lead to systemic complications [5-7].

Diabetes mellitus is an important comorbidity in severe neck infection and has been associated with more complicated clinical courses in deep neck infection cohorts [7,8]. Imaging can clarify the presence, size, and anatomic extent of a collection: ultrasonography is highly sensitive for superficial abscesses, contrast-enhanced computed tomography (CT) is useful for mapping deeper extension and adjacent structures, and magnetic resonance imaging (MRI) provides superior soft-tissue contrast when additional characterization is required [9,10].

We report the surgical management of a large bilobed posterior neck abscess that remained confined to the skin and subcutaneous tissue in an adult with diabetes. The case highlights the role of imaging in excluding deep muscular and cervical-space involvement and the need for timely drainage when symptoms progress despite initial oral antibiotic therapy.

## Case presentation

A 49-year-old man with type 2 diabetes mellitus, hypercholesterolemia, and asthma presented with posterior neck pain that had persisted for 10 days. He described progressive pain and swelling at the cervicothoracic junction. He had previously attended another hospital and received oral amoxicillin-clavulanate; however, the pain worsened and spontaneous discharge developed. Examination revealed an approximately 8 x 6 cm swelling with a draining fistulous opening, more prominent on the right side (Figure 1).

**Figure 1:**
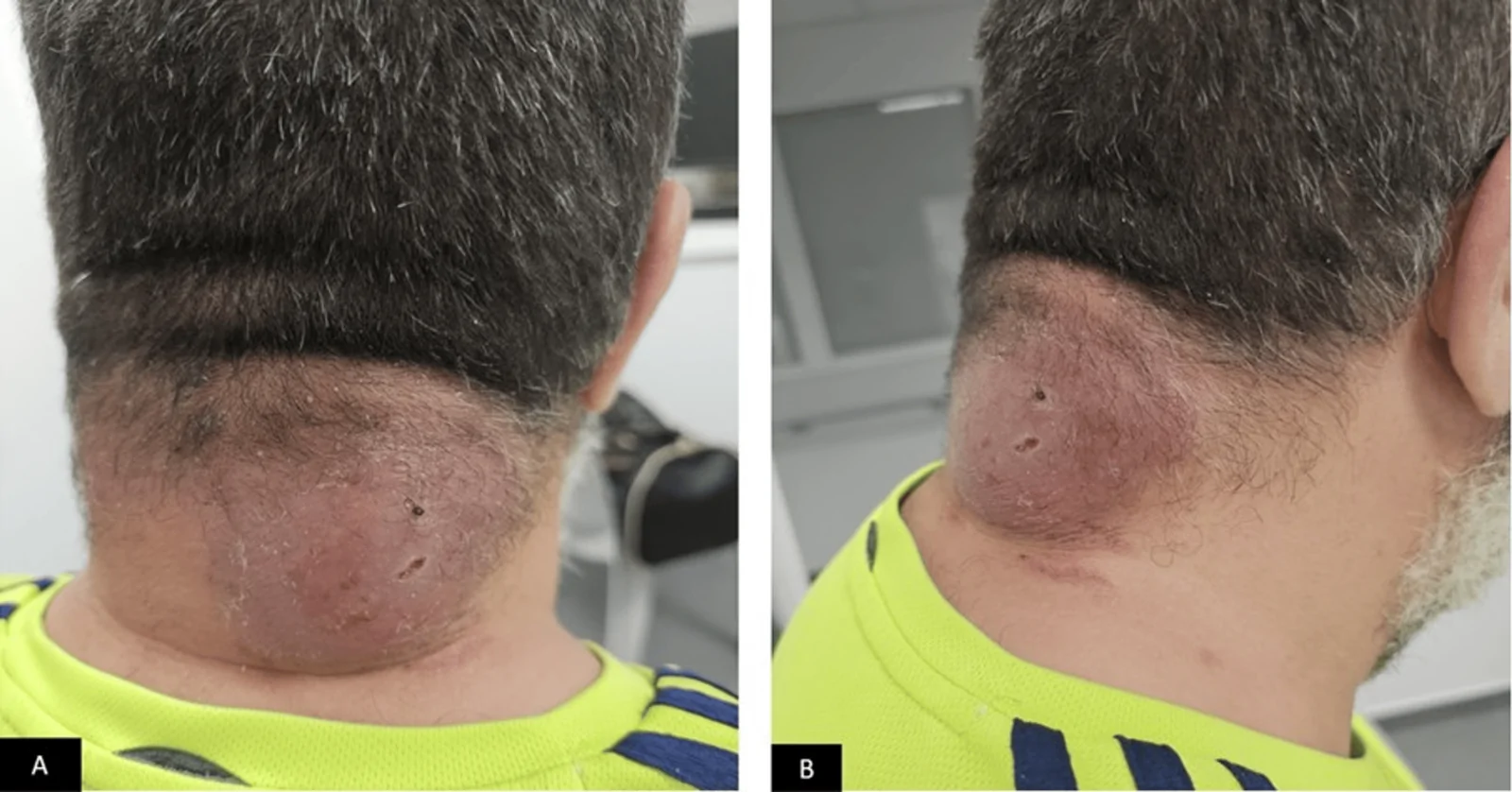
Preoperative clinical appearance of the posterior neck lesion. (A) Posterior view showing a broad area of induration and swelling with cutaneous openings at the nape. (B) Right posterolateral view demonstrating the prominence of the lesion and overlying inflammatory skin changes

The lesion was markedly tender to palpation, with minimal local warmth and erythema. The patient's vital signs were stable, and he was afebrile. Contrast-enhanced neck CT performed in the emergency department demonstrated a bilobed abscess within the posterior subcutaneous tissues, without significant invasion of the paraspinal musculature or extension into the deep cervical spaces. Because of the lesion size, ongoing drainage, worsening symptoms despite oral therapy, and the patient's diabetes, urgent surgical drainage was recommended (Figures 2-4).

**Figure 2:**
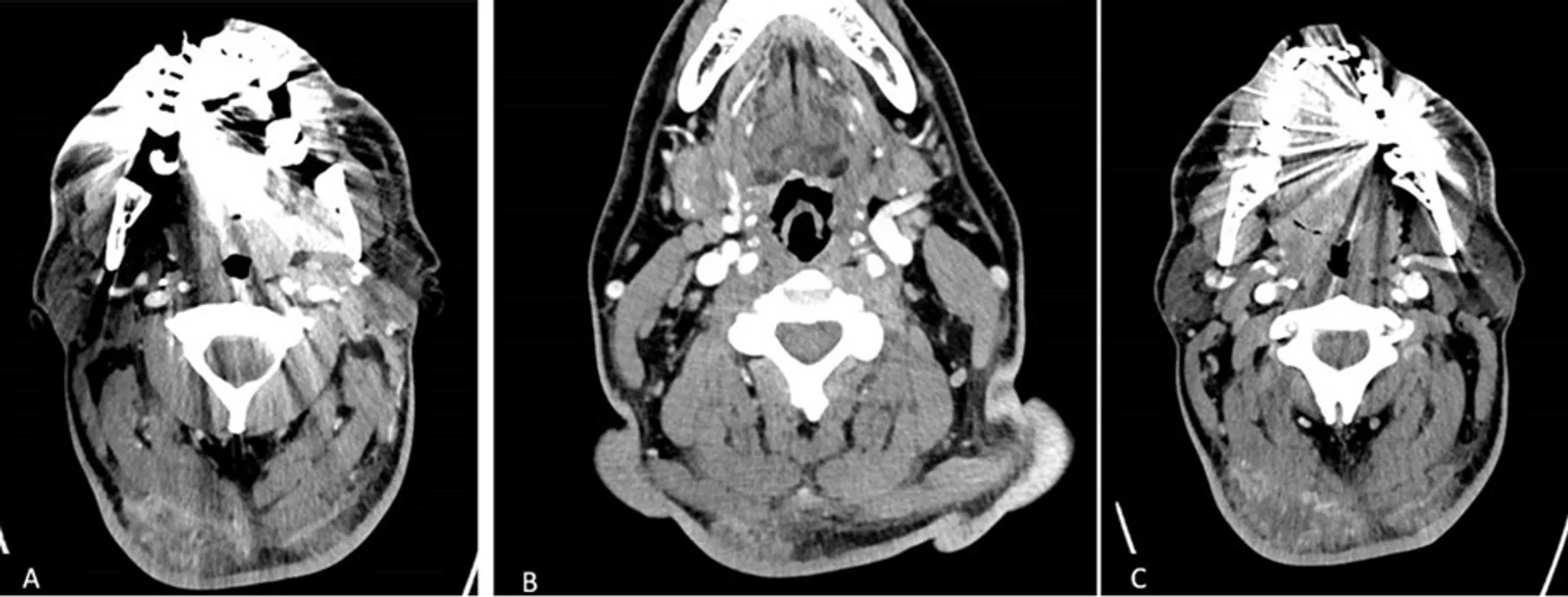
Contrast-enhanced axial CT images at selected cervical levels. (A-C) A bilobed, peripherally enhancing fluid collection is visible within the posterior subcutaneous tissues, with surrounding inflammatory fat stranding. The collection remains superficial to the paraspinal musculature, and no deep cervical-space extension is evident.

**Figure 3:**
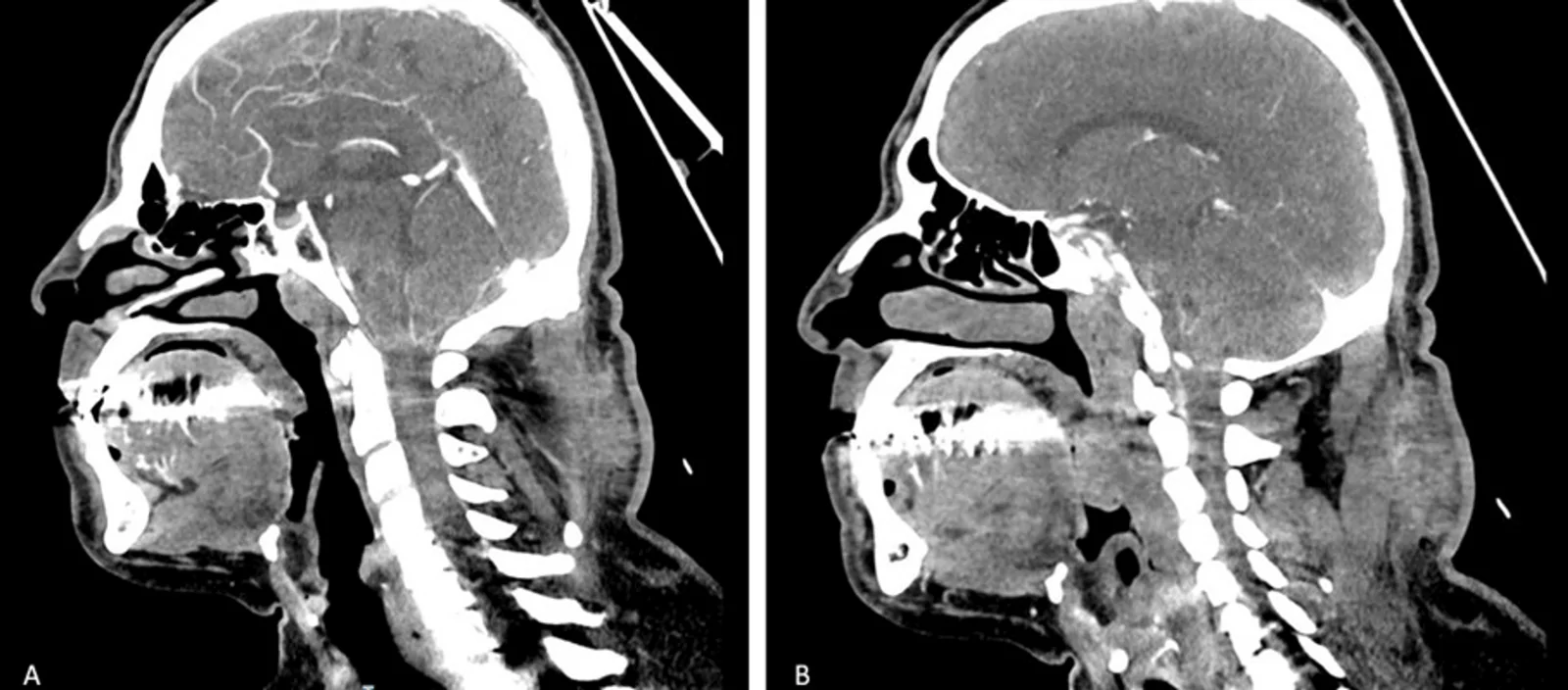
Contrast-enhanced sagittal CT reformations of the posterior neck. (A) A paramedian sagittal image demonstrating the craniocaudal extent of the subcutaneous collection. (B) A more lateral sagittal image showing the lobulated component and its superficial relationship to the posterior cervical musculature.

**Figure 4:**
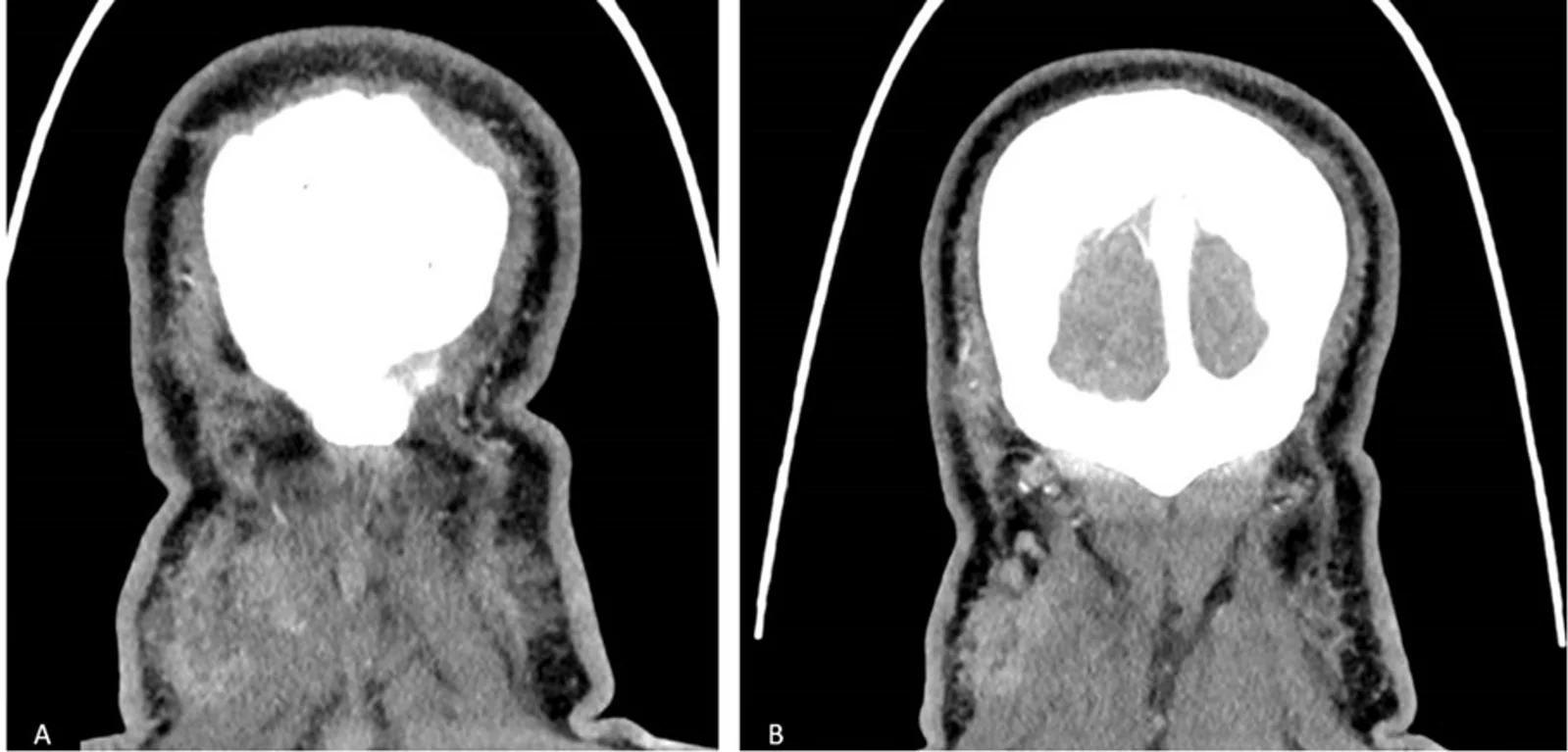
Contrast-enhanced coronal CT reformations of the posterior neck. (A-B) Selected coronal planes demonstrate the transverse extent and bilobed configuration of the collection within the posterior subcutaneous tissues, without extension into the underlying paraspinal muscles.

Under anesthesia, an incision was made over the posterior neck lesion and carried through the skin and subcutaneous tissue. A large amount of dense yellow purulent material was encountered and evacuated. Samples were sent for microbiological culture and pathological examination. The cavity was explored, debrided as required, and irrigated thoroughly with normal saline followed by povidone-iodine solution. The skin and subcutaneous tissues were then approximated over a closed-suction drain.

Dual antimicrobial therapy was initiated on the recommendation of the Infectious Diseases team. Operative cultures yielded *Staphylococcus aureus*, and treatment was subsequently continued according to microbiological findings and specialist advice. The patient remained clinically stable during ward observation. The drain was removed on postoperative day 2, and the patient was discharged with oral antimicrobial therapy and scheduled outpatient follow-up.

## Discussion

This case represents a large posterior neck abscess that was clinically prominent but remained confined to the skin and subcutaneous tissues. This distinction is clinically relevant: superficial abscesses require effective source control, whereas deep neck-space infections may additionally threaten the airway, involve multiple fascial compartments, or extend toward the mediastinum [5,6]. The multiplanar CT findings enabled the surgical team to define the collection and confirm the absence of significant muscular or deep-space involvement before intervention.

Incision and drainage are the cornerstone of treatment for purulent skin and soft-tissue abscesses [1]. Randomized evidence also supports adjunctive anti-staphylococcal antibiotics after drainage in selected patients, while the decision should remain guided by disease severity, comorbidities, local resistance patterns, and culture results [4]. In our patient, the large lesion, progression despite oral amoxicillin-clavulanate, elevated inflammatory markers, and underlying diabetes favored urgent operative drainage, inpatient observation, and Infectious Diseases consultation rather than continued medical therapy alone.

*Staphylococcus aureus* is a major pathogen in purulent skin and soft-tissue infections and is also frequently identified in neck abscess cases [2-4,6]. Culture confirmation in this case allowed treatment to target the recovered organism. Because methicillin susceptibility was not specified in the available case data, the isolate should be reported as *S. aureus *rather than presumed to be methicillin resistant. The duration and route of antimicrobial treatment should likewise be determined by the clinical response, adequacy of drainage, microbiological susceptibility, and specialist assessment rather than by a fixed prolonged course.

Diabetes is associated with impaired host defense and has been linked to more severe or complicated presentations in deep neck infection cohorts [7,8]. Although this patient was afebrile and hemodynamically stable, the absence of sepsis did not exclude a clinically important localized infection. Prompt source control was therefore appropriate, particularly because symptoms had progressed during initial outpatient treatment.

The differential diagnosis of an atypical or chronic cervical mass may include mycobacterial infection. Craniocervical tuberculosis is uncommon but has been reported at unusual sites [11]. Tuberculosis culture was therefore obtained in this patient; however, the acute presentation, operative purulence, and recovery of *S. aureus* supported a pyogenic abscess.

Imaging should be selected according to the clinical question. Ultrasonography is portable and sensitive for superficial collections, whereas CT offers a broader assessment of lesion extent and adjacent deep structures. In a comparative study of superficial abscesses, ultrasonography was more sensitive and CT more specific, although both modalities were clinically useful [9]. MRI provides excellent soft-tissue discrimination and is valuable when deep-space extension, vascular complications, osteomyelitis, or an alternative diagnosis remains uncertain [12]. In this emergency presentation, contrast-enhanced CT rapidly demonstrated both the drainable collection and its confinement to superficial tissues. The principal limitation of this report is its single-patient design and the absence of long-term outcome data; nevertheless, the case illustrates a practical diagnostic and therapeutic approach to an uncommon, anatomically concerning presentation.

## Conclusions

A large posterior neck abscess may produce substantial swelling and drainage while remaining confined to the skin and subcutaneous tissues. Careful examination and timely cross-sectional imaging are essential to define the collection, distinguish superficial disease from deep cervical-space infection, and plan safe source control. In patients with diabetes or clinical progression despite initial oral antibiotics, treatment should not be delayed solely because fever or hemodynamic instability is absent. Prompt incision, drainage, appropriate debridement, microbiological sampling, and culture-directed antimicrobial therapy can achieve effective control while avoiding unnecessary extension of surgery. This case also underscores the importance of describing the precise anatomic compartment involved rather than using the broad term "cervical abscess" without qualification.
